# A Novel Complex A/C/G Intergenotypic Recombinant of Hepatitis B Virus Isolated in Southern China

**DOI:** 10.1371/journal.pone.0084005

**Published:** 2014-01-24

**Authors:** Heling Su, Yan Liu, Zhihui Xu, Shuquan Cheng, Haiyan Ye, Qing Xu, Qingbo Liu, Shuhong Tan, Dongping Xu, Yongming Liu

**Affiliations:** 1 Department of Biochemistry and Molecular Biology, Guilin Medical University, Guilin, Guangxi, China; 2 Viral Hepatitis Research Laboratory, Institute of Infectious Diseases/Liver Failure Medical Center, Beijing 302 Hospital, Beijing, China; 3 Division of Hepatology, The Third People’s Hospital of Guilin, Guilin, Guangxi, China; 4 Guangxi Key Laboratory of Molecular Medicine in Liver Injury and Repair, Guilin Medical University, Guilin, Guangxi, China; University of Cincinnati College of Medicine, United States of America

## Abstract

Hepatitis B virus (HBV) genotypes and subgenotypes may vary in geographical distribution and virological features. Previous investigations, including ours, showed that HBV genotypes B and C were respectively predominant in South and North China, while genotypes A and D were infrequently detected and genotype G was not found. In this study, a novel A/C/G intergenotype was identified in patients with chronic HBV infection in Guilin, a city in southern China. Initial phylogenetic analysis based on the S gene suggested the HBV recombinant to be genotype G. However, extended genotyping based on the entire HBV genome indicated it to be an A/C/G intergenotype with a closer relation to genotype C. Breakpoint analysis using the SIMPLOT program revealed that the recombinant had a recombination with a arrangement of genotypes A, G, A and C fragments. Compared with the HBV recombinants harboring one or two genotype G fragments found in Asian countries, this Guilin recombinant was highly similar to the Vietnam (98–99%) and Long An recombinants (96–99%), but had a relatively low similarity to the Thailand one (89%). Unlike those with the typical genotype G of HBV, the patients with the Guilin recombinant were seropositive for HBeAg. Moreover, a relatively high HBV DNA viral load (>2×10^6^ IU/ml) was detected in the patients, and the analysis of viral replication capacity showed that the Guilin recombinant strains had a competent replication capacity similar to genotypes B and C strains. These findings can aid in not only the clarification of the phylogenetic origin of the HBV recombinants with the genotype G fragment found in Asian countries, but also the understanding of the virological properties of these complicated HBV recombinants.

## Introduction

Hepatitis B virus (HBV) infection remains a global public health problem. More than 240 million people worldwide are chronically infected and at risk of developing progressive liver disease including fibrosis, cirrhosis and hepatocellular carcinoma. About 600,000 people die every year due to the acute or chronic consequences of hepatitis B [1].

HBV belongs to the family of Hepadnaviridae and is a partially double–stranded circular DNA virus. Its approximately 3.2 kb genome encodes four partially overlapping open reading frames (ORFs) for the polymerase (P), core (C), surface (S) and X genes, respectively. Based on sequence divergence in the entire genome exceeding 8% or in S gene exceeding 4%, HBV is currently classified into eight genotypes, designated A to H. In addition, most of these genotypes have been subclassified recently into subgenotypes with distinct virological and epidemiological properties [2]–[5]. Research on HBV genotypes during the last decade has associated the HBV genotypes significantly with the severity of liver disease, clinical outcomes and the response to antiviral therapies [6]–[8]. It has been documented that HBV genotypes or subgenotypes vary in geographical distribution. Genotype A prevails in Europe, Americas and Africa. Genotypes B and C are prevalent in Asia. Genotype D has global distribution, but is predominant in the Mediterranean area. Genotype E spreads commonly in West Africa [9]–[11] . Genotypes F and H are restricted to Central and South America. HBV genotype G is found mainly in the United States and Europe [12]. Moreover, recent studies suggested that recombination events that lead to the emergence of hybrid strains are relatively frequent and of significance in HBV evolution [13]. Recombinant forms between HBV genotypes A and D [14]–[16], B and C [17]–[19], C and D [20], [21], C and G [22] and F and G [23] have been identified in several countries.

China is a country where HBV infection is highly endemic, according to the definition by the World Health Organization. The prevalent HBV genotypes in this country are A, B, C and D, with B and C as major genotypes [24]. In recent years, some recombinant HBV genotypes have been identified in China. These include a C/D intergenotype found in Tibet [25], a novel C/D recombinant genotype found in northwestern China [20], and a complex recombinant genotype X/C found in southern China [26]. The latter harbors an unknown genotype (X) fragment similar in part to genotype G and shows a high similarity (96–99%) to the complex recombinant genotype identified in Vietnam, which is an intergenotype among genotypes A, C, and G [27]. Here we report a complex intergenotypic recombinant of HBV isolated from two unrelated patients with chronic HBV infection in Guilin, a city of Guangxi province in southern China. This recombinant possesses a mosaic fragment of genotypes A, C and G and is highly similar to the Vietnam recombinant.

## Materials and Methods

### Study Subjects

A total of 276 serum samples from patients with chronic HBV infection were collected in Guilin, China. This study was performed in accordance with institutional ethical guidelines and was approved by the Ethics Committee of the Affiliated Hospitals of Guilin Medical University. All patients signed the informed consent and were offered the option to quit participation at any time.

Samples were stored at –70°C until assayed. Two HBV isolates which were suggested to be hybrid strains by initial genotyping were obtained from two unrelated patients of this cohort. Patient 1 was a twenty-four years old female while patient 2 was a thirty-three years old male; both were seen in the Guilin Third People’s Hospital, the Infectious Disease Hospital of Guilin, as outpatients with chronic HBV infection and seropositive for HBsAg, HBeAg and anti-HBc. In addition, the female patient had a normal level of serum alanine transaminase (ALT), while the male patient had an aberrant serum level of 101 IU/L. The HBV DNA viral loads were relatively high, as 2.3×10^6^ IU/ml in female and 2.1×10^6^ IU/ml in the male. Anlysis of HBV replication capacity by quantitation of intracellular HBV replicative intermediates using the method we previously described [28] showed that the levels of the cellular HBV replicative intermediates were 7.5×10^6^ and 3.8×10^6^ IU/ml for two genotype C strains (Genbank accession number: GQ377514 and GQ377517), 2.9×10^6^ and 1.1×10^6^ IU/ml for two genotype B strains (Genbank accession number: GQ377519 and GQ377537), and 5.6×10^6^, 1.3×10^7^ and 5.2×10^6^ IU/ml for three Guilin recombinant strains (Genbank accession number: HQ231883 through HO231885). No statistical significance was observed between different genotypes.

### HBV DNA Genotyping and Cloning

HBV DNA was extracted from 200 µl serum samples using viral DNAout (Tiandz, Inc. Beijing, China), and DNA pellet was resuspended in 200 µl sterile water. As we described before [29], polymerase chain reactions (PCR) were performed to amplify the S gene with 5′- ATCCGCAGGCCATGCAGTGG-3′ (nt 3,194–3,213) as the sense primer and 5′-GTCGTCCGCGGGATTCAGC-3′ (nt 1,458–1,440) as the antisense primer. Standard precautions to avoid contamination during PCR were taken, including a negative control serum included in each run. The obtained amplicons were subjected to direct sequencing for HBV DNA genotyping. The full-length genomic sequence of HBV was amplified according to the method reported by Gunther *et al* [30] with P1 (5′-CCGGATTTTTCACCTCTGCCTAATCA-3′) as the sense primer and P2 (5′-CCGGAAAAAAGTTGCATGGTGCTGG-3′) as the antisense primer. After an initial denaturation at 94°C for 3 min, PCR with 10 µl HBV DNA sample as the template and a PCR kit (Tiandz, Inc. Beijing, China) as the reagents was run for 35 cycles of denaturation at 94°C for 60 s, annealing at 56°C for 40 s and extension at 72°C for 3 min, followed by a final extension at 72°C for 10 min. PCR products were purified with a QIAquick Gel Extraction Kit (Qiagen, Hilden, Germany) and cloned into pGEM-T Easy Vector System (Promega, Madison, USA) according to the manufacturer’s protocol, followed by transformation into JM109 cells (Promega). Positive colonies were selected and sequenced.

### Phylogenetic Analyses

Sequence alignments were carried out using CLUSTAL_X v1.8 software. Phylogenetic trees were constructed by the neighbor-joining method using MEGA software version 5.0 with 1,000 bootstrapped data sets, based on the S gene, pre-S region and full-length genome, respectively. Genetic distance calculation and pairwise distance comparisons using the Kimura two-parameter model were integrated into the MEGA software. Phylogenetic analysis against representative sequences of genotypes A–H was performed. A total of 34 sequences were selected at random from HBV full-length sequences of authentic genotypes A–H, four to five sequences for each genotype, according to Norder *et al* [12] and Yang *et al* [31]. The genome of woolly monkey hepadnavirus was used as outgroup control in the analysis.

### Recombination Investigation

The genotype of HBV was determined using the National Centre for Biotechnology (NCBI) HBV genotyping tool (http://www.ncbi.nlm.nih.gov/projects/genotyping/formpage.cgi). Recombinant genotypes were analyzed with SIMPLOT program version 3.5 (http://sray.med.som.jhmi.edu/SCRoftware/simplot/) [32], which identified phylogenetically informative sites supporting alternative tree topologies. The recombination detection was performed by considering four sequences at a time: one putative recombinant sequence, two reference sequences of original genotype G (GenBank accession no. AB064310) and C (GenBank accession no. AB050018), and one sequence of a known outgroup (woolly monkney; GenBank accession no. AF046996). Each informative site supports one of three possible phylogenetic relationships among the four taxa. Contiguous sites suggesting a single phylogeny were inferred to represent regions between recombination breakpoints. Bootscanning and cluster analysis maximizing *x* ^2^ parameter were used to identify the breakpoints, and *P* values for the subsequent division of the sequence into genotypes were calculated by using Fisher’s exact test.

## Results

### Genotyping based on S gene

The sequences of the entire S-gene of the 276 HBV isolates from the Guilin cohort were analyzed and compared with reference sequences for all eight known HBV genotypes from GenBank database. Of the 276 samples, 170 (61.6%), 104 (37.7%) and 2 (0.7%) were clustered as genotypes B, C and G, respectively.

### Cloning of HBV Full-length Genomes

HBV full-length genome cloning from the female and male patients yielded a total of 14 positive clones, 9 from the female denoted as F-1, F-2, F-3, F-4, F-5, F-6, F-7, F-8, F-9 while 5 from the male denoted as M-1, M-2, M-3, M-4, M-5, most of which had a sequence length of 3,215 nucleotides (nt) except M-3 which had a sequence length of 3,194 nt due to a 21 nt deletion at the 3–23^rd^ nt of its pre-S1 region. The nucleotide sequences of the 14 clones have been submitted to the GenBank database under accession numbers HQ231877–HQ231885 and KF425553–KF425557.

### Analysis of the Characteristics of Genotype G

Given the two isolates were genotyped as genotype G by phylogenetic analysis based on S gene, we used vector NTI suite 8.0 software to further analyze the 14 clones. Typical genotype G is known to be 3,248 nt in length, slightly longer than other HBV genotypes due to an insertion of 36 nt at codon 2 of the C gene [33]. Interestingly, all 14 clones lacked this insert. On the other hand, the codons 2 and 28 of the pre-C region should be stop codons to terminate the translation of HBeAg, but in our 14 clones the codon 2 was a CAA triplet, similar to the B1-89, an HBV genotype G strain reported earlier [33], while the codon 28 was a TGG triplet encoding Trp. Moreover, genotype G should have one amino acid deletion in the pre-S1 region but it was not found in our sequences. These results suggest that none of the 14 obtained clones is a typical genotype G.

### Phylogenetic Analyses

**Figure 1:**
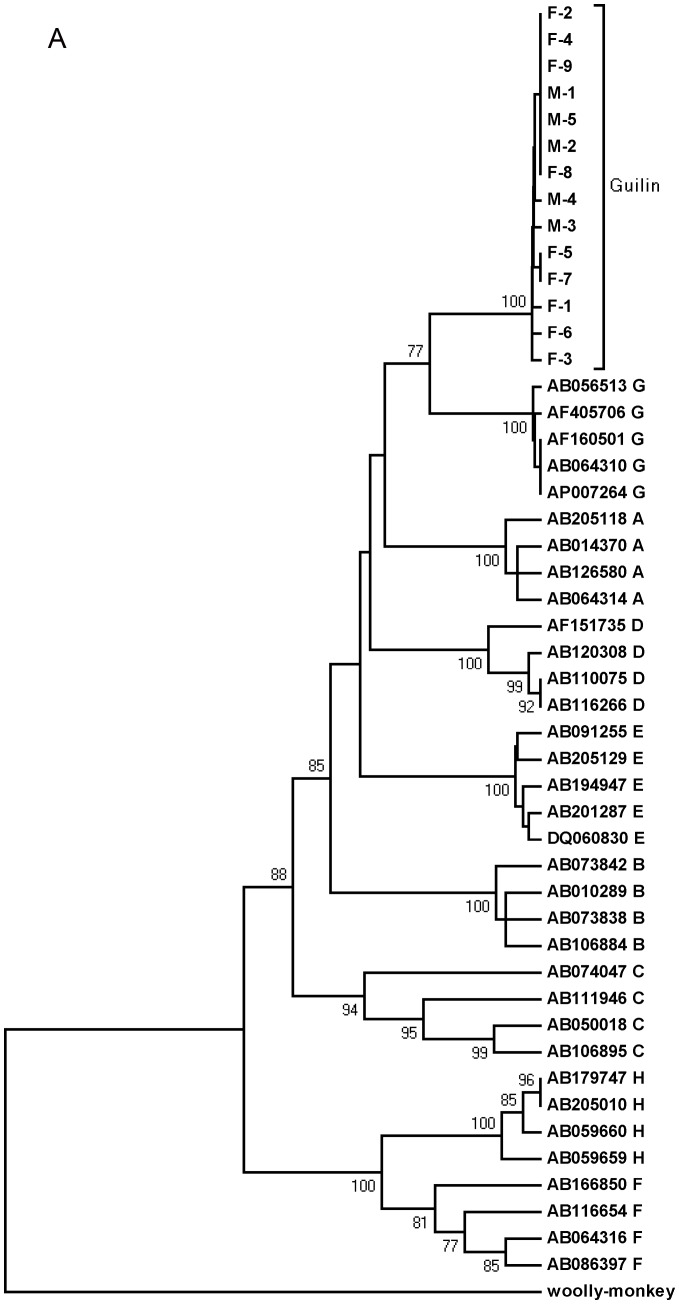
Figure 1. Phylogenetic analysis of the 14 clones derived from the Guilin recombinant compared with reference strains. GenBank accession numbers and clone numbers are showed on each tree, and the genotype is indicated after every accession number. Bootstrap values are showed at each nodes, and only bootstrap values of >70% are indicated. Phylogenetic trees comparing the 14 clones with 34 reference strains representing genotypes A–H, were constructed based on the S gene (A), pre-S region (B) and whole genomes (C). And the phylogenetic tree was constructed by comparing the Guilin recombinant with the Vietnam, Thailand and Long An recombinants as well as HBV C subgenotype based on the whole genomes (D).

While phylogenetic analysis based on the S gene suggested that the 14 clones obtained were genotype G with a 77% bootstrap value (Figure 1A), extended analysis based on the pre-S region and the whole genomic sequence showed that they were genotype A with a 71% bootstrap value (Figure 1B) and genotype C with a 100% bootstrap value (Figure 1C), respectively, which dovetails the results from using the NCBI HBV genotyping tool (http://www.ncbi.nlm.nih.gov/projects/genotyping/formpage.cgi). Similar results were also obtained by pairwise comparison between the sequences of the 14 clones and known genotypes using the S gene, pre-S region and full-length genome: in the case of the S gene, it was most similar to genotype G (2.9%±0.6%, difference); in the case of the pre-S region, it was more similar to genotype A (10.0%±1.4%, difference); and in the case of the full-length genome, it was more similar to genotype C (8.0%±0.4%, difference) (Table 1). Taken together, the Guilin HBV recombinant from which the 14 clones were obtained is considered as an A/C/G intergenotype.

**Table 1:** Nucleotide distances between the Guilin HBV recombinant and other reference genotype strains*.

% Nucleotide distance (mean±SD)			
Genotype	S gene	Pre-S region	Complete genome
A	3.9±0.7	10.0±1.4	8.4±0.4
B	4.8±0.8	16.3±1.9	10.1±0.5
C	6.3±0.9	11.8±1.5	8.0±0.4
D	3.9±0.7	19.2±2.1	10.8±0.6
E	4.0±0.7	17.0±1.9	10.5±0.5
F	6.7±0.9	24.5±2.3	14.8±0.6
G	2.9±0.6	15.7±1.9	11.2±0.6
H	7.0±0.1	21.3±2.2	14.6±0.6

The reference genotype strains used in the table are the same as in Figure 1C.

### Identification of the Putative Recombination Sites

**Figure 2:**
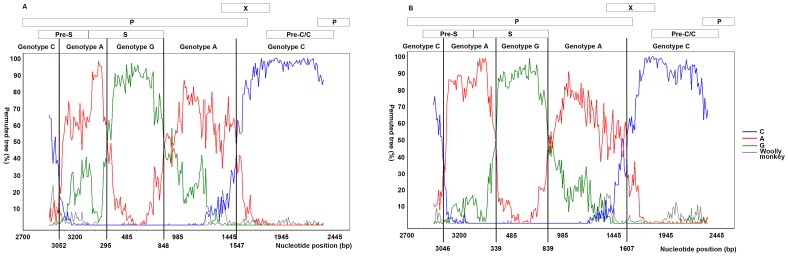
Bootscan analysis demonstrating the complex recombination among genotypes A, C and G in the Guilin recombinant. The isolate from the female patient (A) and the isolate from the male patient (B) were subjected to bootscan analysis over the complete genome using the SIMPLOT program with a 500 bp window size, 10 bp step size and 100 bootstrap replicates, using gap-stripped alignments and neighbor-joining analysis, and were compared with three representative HBV genotypes: A (GenBank accession no. AB126580), C (GenBank accession no. AB050018) and G (GenBank accession no. AB064310). Woolly monkey was a known out-group (GenBank accession no. AF046996). Analysis was stared from nt 2700.

Determination of the breakpoints of genomic recombination in the 14 clones with SIMPLOT program suggested a recombination among genotypes A, C and G in the Guilin recombinant, verifying the results of phylogenetic analyses. Bootscanning showed that in the genomes of the clones derived from the female patient, the 3,052–295^th^ and 848–1,547^th^ nt regions were closely related to genotype A, whereas the 295–848^th^ and the 1,547–3,052^nd^ nt regions were closely related to genotype G and C, respectively. Similarly, in the HBV genome from the male patient, the 3,046–339^th^ and 839–1,607^th^ nt regions were closely related to genotype A, whereas the 339–839^th^ and 1,607–3,046^th^ nt regions were closely related to genotypes G and C, respectively (Figure 2A, 2B). Bootscan analysis also revealed that most (nt 2,848–155) of the pre-S region, most (nt 155–833) of the S gene, and most of the whole genome belonged to genotypes A, G and C, respectively. These results were consistent with phylogenetic analyses.

### Comparison of HBV Recombinants

We further compared the Guilin recombinant with the Vietnam, Long-An and Thailand recombinants that had features in common, i.e., bearing one or two genotype G fragments and being identified in Asian countries. Sequence alignment showed that the Guilin recombinant was highly similar to the Vietnam (98–99%) and Long An ones (96–99%), but had a relatively low similarity to the Thailand recombinant (89%). Table 2 presents the positions of breakpoints and the arrangements of the genotype fragments of the four recombinants and exhibits that these four have different breakpoint positions and arrangements of genotype fragments. Phylogenetic tree construction based on the full-length genomes of the four recombinants with subgenotypes C1–C16 as the reference sequences [34] revealed that the Guilin, Vietnam and Long An recombinants were all clustered on a branch from subgenotype C1–C16, supported by a 100% bootstrap value (Figure 1D), while Thailand recombinant clustered on a different branch.

**Table 2:** The breakpoint positions and the arrangements of the genotype fragments of the Guilin, Vietnam, Long An and Thailand recombinants.

Recombinant location	breakpoint positions	the arrangements of the genotype fragments
Guilin	3050–330; 330–850; 850–1600; 1600–3050	A; G; A; C
Vietnam	1–396; 396–666; 666–872; 872–1104; 1104–3215	A; G; C; G; C
Long An	1–1250; 1250–1670; 1670–3100	G; unknown genotypes; C
Thailand	1–1860; 1860–2460; 2460–3215	G; C; G

The analysis of the nt and deduced amino acid sequences of these recombinants showed that the pre-S/S gene of the Vietnam and Long An recombinants, but not the Thailand one, encodes distinctive conserved amino acids such as His^56^, Ala^60^, Asn^87^, Val^90^, Val^91^, Ile^136^, and Lys^198^, which do not belong to genotypes A, C, or G. Moreover, while the recombinant from the female patient had these distinctive conserved amino acids, the male patient’s recombinant showed a small difference, having Ala^90^ rather than Val^90^.

In addition, the serological subtypes of the Guilin, Vietnam and Thailand recombinants were adw. In contrast, 90% of the serological subtype of Long An recombinant was adw, and 10% was adr.

## Discussion

The most interesting finding in this study is the identification of two HBV hybrid strains from two unrelated patients as a novel complex intergenotype of genotypes A, C and G. It is known that the genotype G of HBV has been detected mainly in the United States and Europe [12], commonly co-infects with HBV genotype A or H [33], [35], and has not been reported in China so far. In recent years, however, several HBV recombinant genotypes with one or two genotype G fragments have been identified in Asian countries including China. In 2005, the first C/G recombinant genotype was identified in Thailand [22] and, based on SIMPLOT analysis, most of its genomic sequence belongs to genotype G. In 2008, another HBV recombinant genotype with the genotype G fragments was identified in Vietnam [27], which is an intergenotype of genotypes A, C and G and was denominated as genotype I by the authors. However, this new denomination was not accepted by other experts in HBV phylogeny because the genetic distance from genotype C is within the 8% limit [36]. More recently, Fang *et al* isolated another complex intergenotype in Long An county of southern China [26], which was an intergenotype between genotype C and an unknown genotype (X) with some similarity to genotype G. We find that the Guilin, Long-An, Thailand and Vietnam recombinants have different breakpoints and arrangements of genotype fragments (Table 2). Nevertheless, the Guilin recombinant is highly similar to the Vietnam and Long An ones but dissimilar to the Thailand one. Phylogenetic analysis based on the full-length genomes of all four recombinants using subgenotypes C1 through C16 as the reference sequences revealed that the Guilin, Vietnam and Long An recombinants are clustered on the same clade, while the Thailand recombinant is clustered on a different branch. In addition, the pre-S/S gene of the Guilin, Vietnam and Long An recombinants encodes distinctive conserved amino acids that do not belong to genotypes A, C, or G, while the Thailand one has none of these amino acids. Therefore, the Guilin recombinant is considered as a new subgenotype C rather than a new genotype and to belong the same subgenotype as the Vietnam and Long An ones.

Although the Guilin recombinant bears a genotype G fragment, it does not show the characteristics of genotype G. It is known that patients with genotype G should lack HBeAg because the two stop codons at codons 2 and 28 in the pre-C region terminate its translation. Since HBeAg is an immunotolerogen required for the establishment of persistent infection, lack of its expression is the major cause for the rare occurrence of genotype G monoinfection [37]. Given that the fragment of genotype G of the Guilin recombinant is located at nt 330–850 in the HBV genome and its C gene and pre-C region are genotype C, we predict that the recombinant should express HBeAg. Indeed, the two patients infected with the recombinant were seropositive for HBeAg. Moreover, a relatively high HBV DNA viral load (2.1–2.3×10^6^ IU/ml) was detected in the two patients, and the measurement of viral replication capacity showed that the Guilin recombinant strains had a competent replication capacity similar to genotypes B and C strains.

It is puzzling that the unusual A/C/G intergenotypic recombinant was found in Guilin, because genotype A is a rare one in the Chinese population and genotype G has not been reported so far in China. Guilin City and Long-An County belong to Guangxi province in southern China, both bordering the northern part of Vietnam where the Vietnam recombinant was identified. Close geographical relations combined with high sequence similarities within Guilin, Long An and Vietnam recombinants suggest the close relations within the three recombinants. Fang *et al* inferred, based on bioinformatic analysis, that the Long An recombinant probably originated in southern China and later spread to Vietnam and Laos [26]. Our data support the inference but it awaits further investigation.

Phylogenetic analysis of the S gene in the HBV genotypes of the 276 patients in the Guilin cohort shows that the dominant HBV genotypes in the patients are B and C, accounting for 61.6% and 37.7%, respectively, with the remaining 0.7% as a recombinant genotype. An earlier nationwide study reported that the prevalence of the HBV genotypes A, B, C and D were 1.2%, 41.0%, 52.5% and 4.3%, respectively in a Chinese population [24]. We reported previously that the prevalence of HBV genotypes B, C and D were 14.6%, 84.2% and 1.2%, respectively in North China [38]. In this Guilin cohort, genotypes A and D have not detected while genotype B is the most dominant. These results supplement the HBV genotype information in Guilin area and suggest a geographic divergence of the prevalence of HBV genotypes in different regions of China.

In conclusion, this study identifies a new complex A/C/G intergenotype of HBV from two unrelated patients and presents the data of the prevalence of HBV genotypes in the patients with chronic HBV infection in Guilin. These findings contribute not only to the clarification of the phylogenetic origin of HBV recombination, especially the phylogenetic origin of the HBV recombinants with the genotype G fragment found in Asian countries, but also to our understanding of virological properties of these complicated HBV recombinants.
